# Prevalence of Elder Abuse and Sub-types in South Asia: A Systematic Review and Meta-analysis

**DOI:** 10.1093/geroni/igaf122.2045

**Published:** 2025-12-31

**Authors:** Aman Shrestha, Saruna Ghimire, Krishna Prasad Sapkota, Isha Karmacharya, Indrajit Ghosh, Ahmed Danquah, Nicole Shelawala, Emily Gorman

**Affiliations:** University of Maryland, Baltimore County, Baltimore, Maryland, United States; Miami University, Oxford, Ohio, United States; Miami University, Oxford, Ohio, United States; Miami University, Oxford, Ohio, United States; Eli Lilly and Company, Indianapolis, Indiana, United States; Miami University, Oxford, Ohio, United States; University of Maryland, Baltimore County, Baltimore, Maryland, United States; University of Maryland, Baltimore County, Baltimore, Maryland, United States

## Abstract

Elder abuse is a critical public health and human rights issue, particularly in South Asia, where patriarchal norms, family-centered caregiving and inadequate institutional support exacerbate the issue. Despite global focus, regional data on elder abuse in South Asian countries are limited. This systematic review and meta-analysis estimated the prevalence of community-based elder abuse and its subtypes among older adults (≥60 years) in South Asia and explored the gender differences in abuse experiences. A comprehensive search of six databases and gray literature identified 33 relevant studies from Bangladesh, India, Nepal, Pakistan, and Sri Lanka. Data were extracted using Covidence, and study quality was assessed with the modified Newcastle-Ottawa Scale. Meta-analyses were conducted using random-effects models, with heterogeneity evaluated via the I² statistic. Most studies were from India (n = 21) and Nepal (n = 7), with an overall elder abuse prevalence of 31.8%, ranging from 25.8% in India to 49.9% in Nepal. Women experienced higher abuse rates (33.0%) than men (24.3%). Caregiver neglect and psychological abuse (around 20% each) were the most common, disproportionately affecting women. Physical abuse (3.9%), financial exploitation (8.0%), and sexual abuse (0.7%) were less frequent, with women experiencing higher abuse in all categories except sexual abuse. This study underscores the significant prevalence of elder abuse in South Asia, with women being disproportionately affected, emphasizing the urgency for culturally appropriate interventions. Targeted policies and community-driven initiatives are essential to combat elder abuse, enhance eldercare, and safeguard vulnerable populations in the region.

